# Supplementary material for: Arrangement and symmetry of the fungal E3BP-containing core of the pyruvate dehydrogenase complex
Source: Nat Commun. 2020 Sep 16;11:4667. doi: 10.1038/s41467-020-18401-z (PMC7494870; doi:10.1038/s41467-020-18401-z)
Supplement: Supplementary file 1 — Supplementary Information [file 41467_2020_18401_MOESM1_ESM.pdf]

Supplementary information to ”Arrangement and symmetry of the fungal E3BP-containing core of thePyruvate Dehydrogenase Complex”

B. O. Forsberg[1], S. Aibara[1,2] , R. J. Howard[1], N. Mortezaei[1,3], and E. Lindahl[1,4,*]

[1]Department of Biochemistry and Biophysics, Science for Life Laboratory, Stockholm University, 17165 Solna, Sweden

[2]Current address: Department of Molecular Biology, Max Planck Institute for Biophysical Chemistry, 37077 Göttingen, Germany

[3]Current address: Vironova AB, 11330 Stockholm, Sweden

[4]Department of Applied Physics, Swedish eScience Research Center, KTH Royal Institute of Technology, 17168 Solna, Sweden

[*] Correspondence: erik.lindahl@dbb.su.se

Supplementary methods

Processing of native PDC

139 PDC particles were picked manually from nine micrographs and aligned by 2D-classification. The resultant class average was used to pick 350k candidate particles from 4867 micrographs. A subset of 139k particles was selected based on two sequential rounds of 2D-classification. An icosahedral initial reconstruction was generated in RELION. An icosahedral refinement was next performed under icosahedral symmetry to center particles. The magnification was adjusted, determined by rescaling the map to correlate maximally with the reconstituted E2-PX30 subcomplex which was used to build the atomic model.

Following re-extraction, a 3D-classification of 10 classes under icosahedral symmetry was used to further identify 70k particles that appeared to be of higher quality, based on visual inspection. This subset was refined to 4.1 Å (EMDB 11266), and used for all subsequent analysis. Classification identified two tetrahedral and one over-symmetrized class, used as seeds for a multi-reference classification (described below). Subsets of 30% each of the tetrahedral classes were found and individually refined to 4.2 Å and 4.3 Å respectively. As post-processing appeared to over-sharpen the lower-resolution interior, local-resolution filtering was conducted in RELION (EMDB 11267/8).

Processing of tE2+PX30 reconstituted subcomplex

225k particles were picked automatically from 5675 micrographs using WARP[47]. A consensus refinement was used to center and generally align particles. These (3D-)alignments where used to perform 2D-classifications without further orientation alignment, from which 213k particles were selected. These particles produced a 3.5 Å reconstruction under icosahedral

symmetry. Following CTF-refinement and beam-tilt correction, the icosahedral reconstruction reached 2.9Å (EMDB 11270). The final magnification was adjusted by rescaling the map to optimize covalent geometry metrics during refinement in PHENIX.

Processing of tE2/PX261 reconstituted subcomplex

104 particles were manually picked from 9 micrographs, and averaged by 2D-classification to generate picking templates in RELION. 356,000 particles were then picked from 4063 micrographs. Following 2D-classification, 342,344 particles were subjected to 3D-classification. Repeated attempts to identify a tetrahedral class of particles were unsuccessful, but the interior clearly displayed elevated density consistent with PX over-symmetrization and icosahedral sup-symmetry. Ultimately, 306,327 particles were selected for high-resolution refinement under icosahedral symmetry, and refined to 3.1 Å (EMDB 11271).

Processing of tE2 core complex

134 particles were picked manually from nine micrographs. These were averaged by 2D-classification and used as references for automated picking in RELION. Almost 25,000 particles were picked from 344 micrographs, followed by cleaning through multiple rounds of 2D-classification. 3D-classification under icosahedral identified further data which did not appear to represent E2-core particles. 7347 particles belonged in classes representing intact PDC core particles. These were refined to 5.4 Å, which after refinement of CTF and beam-tilt improved to 4.3 Å.

Supplementary tables

	Stock [mM]	Use [μl]	Final [mM]
HEPES pH7.5	20	800	17.0
NAD	50	50	2.5
Na-Pyruvate	100	20	2.0
Na-CoA	10	20	0.2
TPP	10	20	0.2
MgCl	50	20	1.0
PDC	2.2 mg/ml	10	23 μg/ml
Total		940	

Supplementary Table 1: **Reactants in activity assay**

Dataset	Native	E2	tE2+PX30	tE2/PX261
Grid		Quantifoil R1.2/1.3	Quantifoil R1.2/1.3	Quantifoil R1.2/1.3
Carbon support	Yes	No	No	No
Microscope	FEI Talos	FEI Talos	FEI Krios	FEI Krios
Detector	Falcon 2	FEI Falcon 3	Gatan K2 GIF	Gatan K2 GIF
Voltage	200 kV	200 kV	300 kV	300 kV
Magnification				
Nominal	114k (1.23Å/pix)	(1.23Å/pix)	165k (0.83Å/pix)	165k (0.86Å/pix)
Calibrated	112k (1.25Å/pix)	-	163k (0.85Å/pix)	-
Mode	Counting	Linear	Counting	Counting
Gain normalized	Yes	Yes	Yes	Yes
Micrographs	4867	344	5675	4063
Total dose	35.0 e/Å^2	33.5 e/Å^2, 4s	27.3 e/Å^2, 4s	31.4 e/Å^2, 4s
Fractions	19	39	20	32

Supplementary Table 2: **Datasets collected.** Calibration of magnification was performed for reconstructions with associated atomic models. For tE2+PX30 calibration was performed by PHENIX-refinement against multiple rescaled maps. The best agreement in terms of overall refinement statistics was chosen. For native, the map was rescaled to correlate maximally with the calibrated tE2+PX30 map.

Dataset	Native			E2	tE2+PX30	tE2/PX261
Interior config	over-sym	X4S	Y4S	empty	over-sym	over-sym
Particles	69 538	18 558	21 129	7 347	129 588	306 327
EMDB identifier	11266	11267	11268	11269	11270	11271
Symmetry (order)	I2 (60)	T(12)	T(12)	I2 (60)	I2 (60)	I2 (60)
Sharpening B-factor	-260	-210	-200	-200	-130	-170
Resolution [Å](FSC @ 0.143)	4.1	4.4	4.3	4.4	2.9	3.1
PDB identifier			6ZLM		6ZLO	
Chains built			60 E2 + 12 PX-BH		60 E2	
bond RMSD						
Length [Å] (#$>4\sigma$)			0.004 (146)		0.004 (0)	
Angles [°] (#$>4\sigma$)			0.650 (0)		0.747 (0)	
isotropic ADP (min/max/mean)			56.22/142.49/86.48		9.71/80.00/35.51	
Molprobity score			1.08		1.20	
Clash score			2.24		2.95	
Ramachandran [% O/A/F]			0.00/2.35/97.65		0.00/2.61/97.39	
Rotamer outliers [%]			0.00		0.52	
model-map FSC [Å] (0/0.143/0.5)						
masked			4.1/4.2/4.4		2.8/2.8/2.9	
unmasked			4.2/4.3/4.4		2.8/2.9/3.0	

Supplementary Table 3: **Reconstructions.** tE2+PX30 refers to reconstitution of separately expressed components, whereas tE2/PX261 was a subcomplex of co-expressed components. The E2 core was built ab-initio into the PX30 reconstituted subcomplex reconstruction. The model for the native Y4S model was based on the same E2-core model, placing further small helical poly-Ala section into the density corresponding to the PX-BH. The records of these residues were refined as UNK residue type. .

Supplementary figures

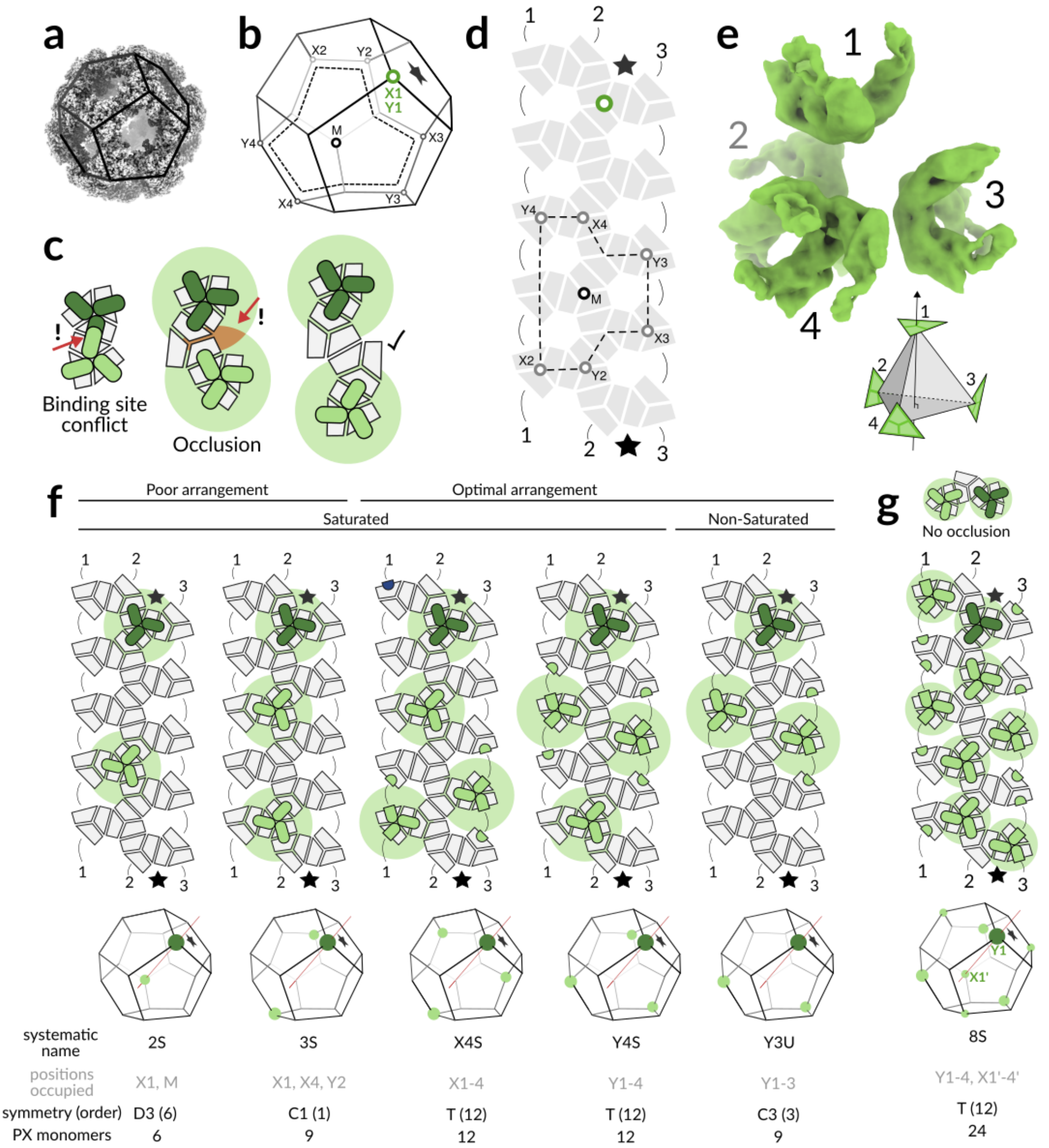

Supplementary Figure 1: **Configurations of the fungal PDC interior.** **a**, The PDC core is a dodecahedron, where the vertices are E2 trimers, and edges are points of contact between adjacent such trimers. **b**, A sketch illustrates important vertices for the subsequent arrangement of PX interior to the PDC core. Each PX trimer occupies one E2 trimer in the sense that their threefold symmetry axes coincide and that the PX trimer binds all edges connecting the E2 trimer vertex. **c**, This means that adjacent vertices cannot be PX-occupied, since the edge is already occupied. The volume occlusion by PX also imposes a steric condition. This is illustrated schematically as a circular background centered on each PX trimer. It is evident from examination of 3D reconstructions that PX occupancy at vertices separated by exactly one intermediate vertex results in clashes of the PX oligomer-occluded volume. Rather, each PX-occupied vertex must be separated from any other by at least two edges. **d**, We unfold the assembly of E2-trimers (which forms the PDC core) to easily illustrate the possible configurations of PX in the E2 core interior. **e** The isolated core-interior density of the tetrahedral reconstruction shows four threefold oligomers, which is of primary interest. **f** However, we may enumerate many possible interior configurations of trimeric density. The points corresponding to those in panel **b** are indicated, and one face is marked with a star for clarity. Numbered lines indicate edges that are broken by unfolding the dodecahedron into this 2D-schematic. Without loss of generality, we can choose one E2-trimer to always be PX-associated. All further consideration regards which additional E2-trimers may be occupied. There is exactly one configuration wherein just two PX trimers are placed such that no further PX trimer can be placed, hence saturated (2S, S=Saturated). We are able to construct at least one 3S configuration, and there are exactly two 4S configurations (X4S and Y4S). The latter two correspond to classes X and Y found in the native PDC data. We also show one example of an unsaturated configuration (Y3U, U=unsaturated), corresponding to a likely configuration in the non-tetrahedral class N. The resultant symmetry is also listed for each displayed configuration along with the order of the symmetry in parenthesis, as well as the number of PX monomers per core. **g**, If the oligomerization of PX interior to the core does not result in a volume occlusion as shown in panel **c**, the optimal arrangement is still tetrahedral, but with a doubled stoichiometry. This 8S configuration is equivalent to a superposition of configurations Y4S and a rotated X4S.

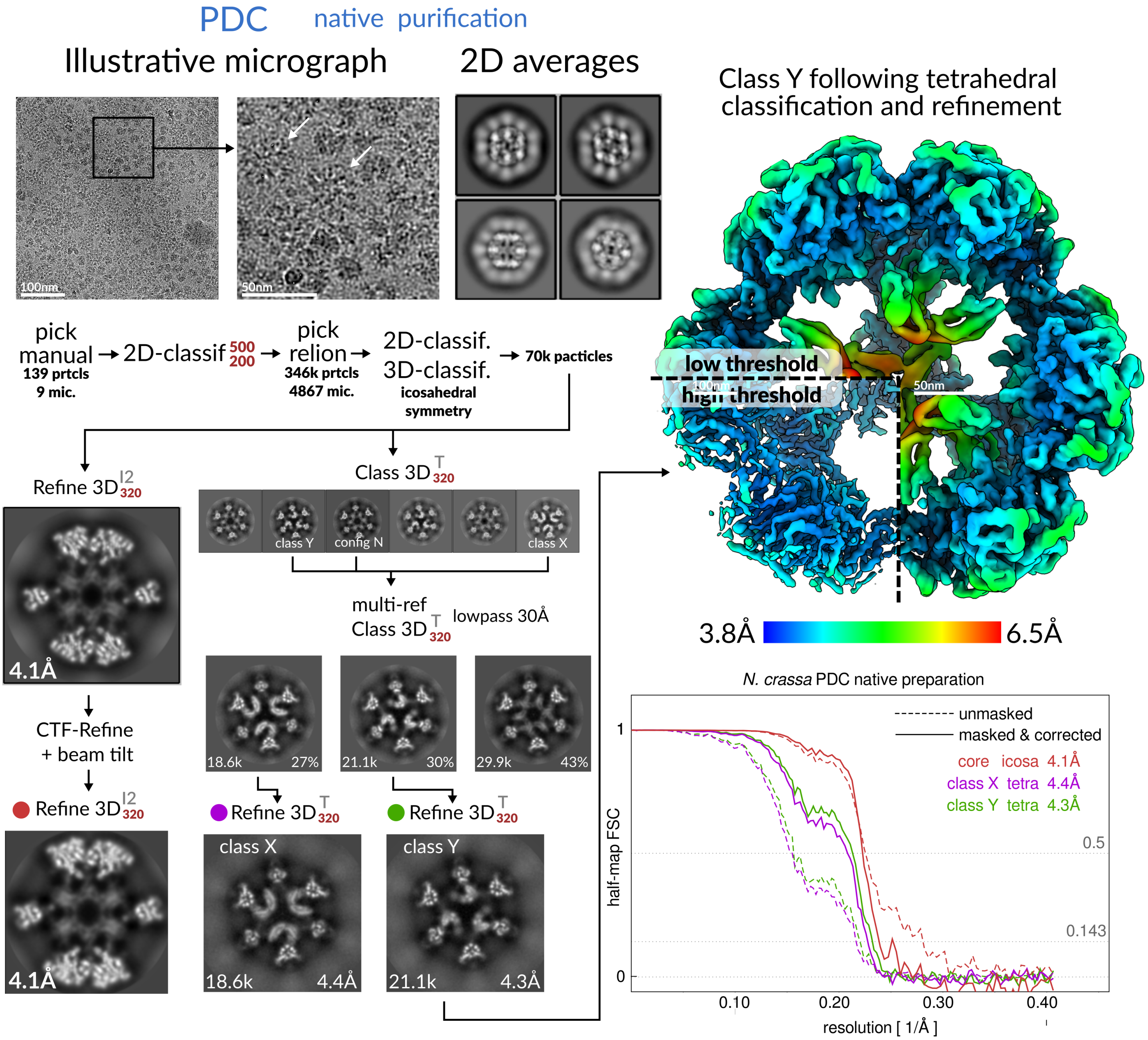

Supplementary Figure 2: **Processing of the native PDC.** Multi-reference classification was conducted by providing three input references, corresponding to the three indicated class reconstructions X, Y and N. The full dataset (70k particles) was thus re-classified to assess the distribution among classes. Corrected FSC refers to the adjustment of the half-map according to over-fitting identified by phase-randomization beyond FSC=0.8.

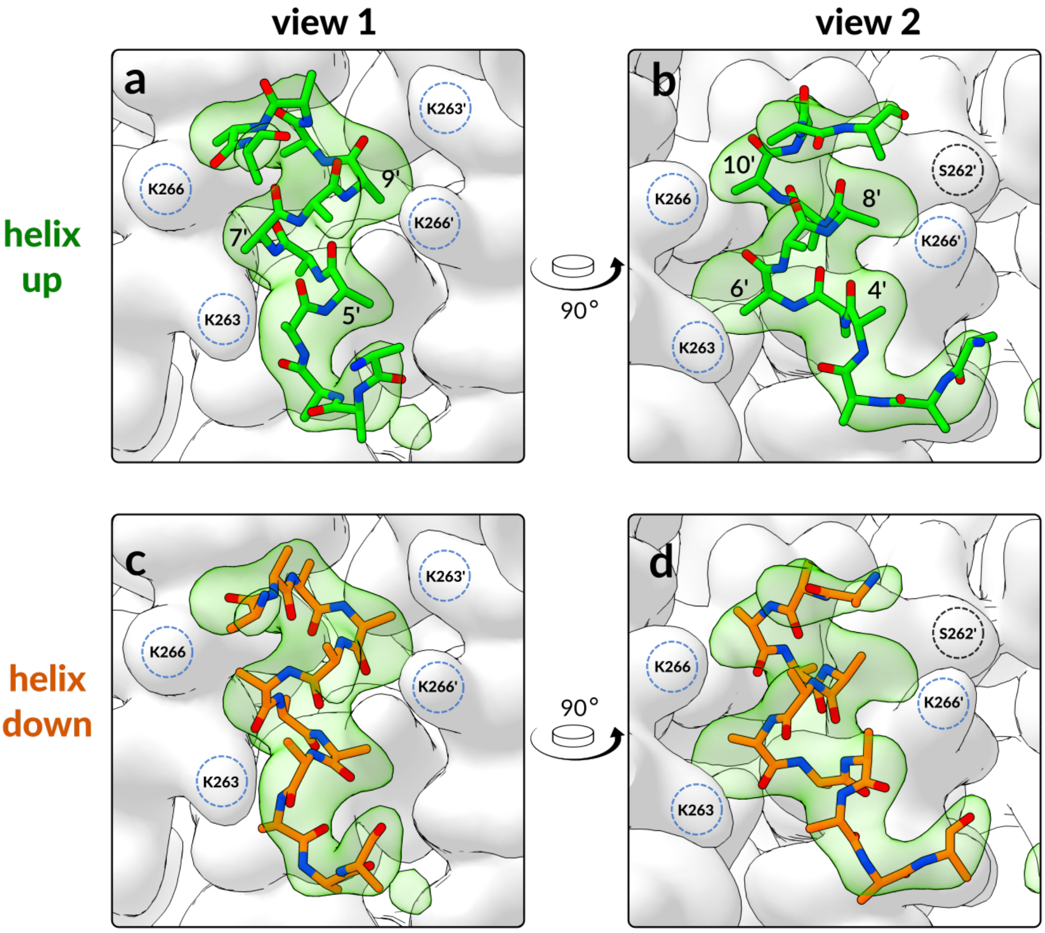

Supplementary Figure 3: **PX binding helix.** The tetrahedral symmetry imposed to reconstruct the tetrahedral classes X and Y results in a break of the strict twofold symmetry of the E2 binding pocket where the PX binding helix (BH) binds, but the pocket itself which is composed of helix-2 (H2) of E2 still appears completely symmetrical, with no detectable induced fit. Even so, the BH appears to bind with a bias for one orientation, apparently enforced by the handedness of the oligomer. Its orientation is somewhat ambiguous, and so both possible orientations are built and considered. The limited resolution prevents modeling of side-chains, which in turn prevents a model-to-map cross-correlation from discriminating which orientation is more likely. However, based on the direction of weaker and residual side-chain densities, we nonetheless find one direction (● panels **a+b**), is more likely than the other (● panels **c+d**). The density is identical in all panels. This interpretation leads to a stronger density connecting the BH N-terminus to the PX-oligomer, indicating that the BH is C-terminal to at least part of the oligomer domain.

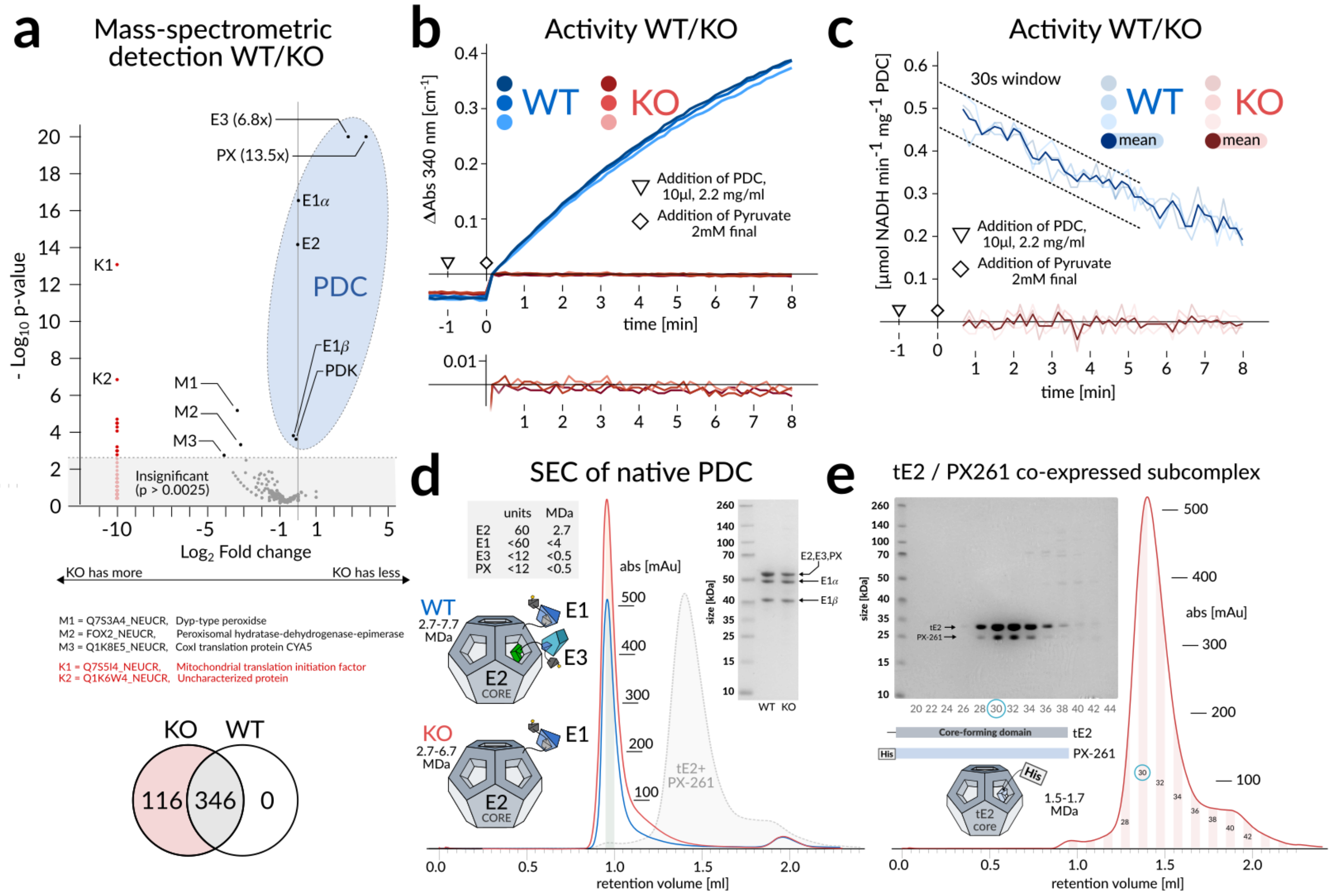

Supplementary Figure 4: **Size exclusion, activity and mass spectrometry.** **a**, Mass spectrometry (MS) confirms that the knockout causes concomitant loss of E3, with no other changes attributable to the PDC-interaction. Some proteins appear to be up-regulated in response to PX-knockout. We cannot rationalize why these would be co-purified with the PDC through SEC, other than their general abundance and the sensitivity of the MS analysis. **b**, Time-resolved kinetics of the purified PDC confirms the detrimental effect of PX knockout on PDC function. Purified complex and pyruvate were added at t=-1 min and t=0 respectively, to a cuvette containing all other necessary reactants (see methods, Supplementary Table 1). **c**, Same as in panel **b**, but with the rate of change averaged over 30s windows following initiation of the reaction. PDC function appears completely eliminated in the KO, attributable (but not necessarily limited) to loss of E3 as shown in panel **a**. The activity appears to decrease rapidly, so the WT activity is extrapolated to $\sim$0.5μmol NADH min^{-1}mg^{-1} PDC at t=0. **d**, Size exclusion chromatography (SEC) of wildtype and knockout PDC indicate similar native size, which is expected given the small relative contribution of PX and E3. An SDS-gel of the peak fraction (shaded area) displays a weakened band where E2, PX and E3 overlap. **e**, A co-expressed subcomplex shows that affinity-purified PX261 retains a high affinity to the PDC core, with no detectable unbound PX following SEC. This may indicate a stoichiometric deficit of PX261 and subsequently unsaturated core complexes, which could rationalize why tetrahedral classes are more elusive in this cryo-EM dataset, despite the obvious occupancy of the core interior. This is corroborated by the observation that expression of PX261 is greatly hampered in the absence of co-expressed E2 or tE2, indicating that it is sensitive to degradation or proteolysis.

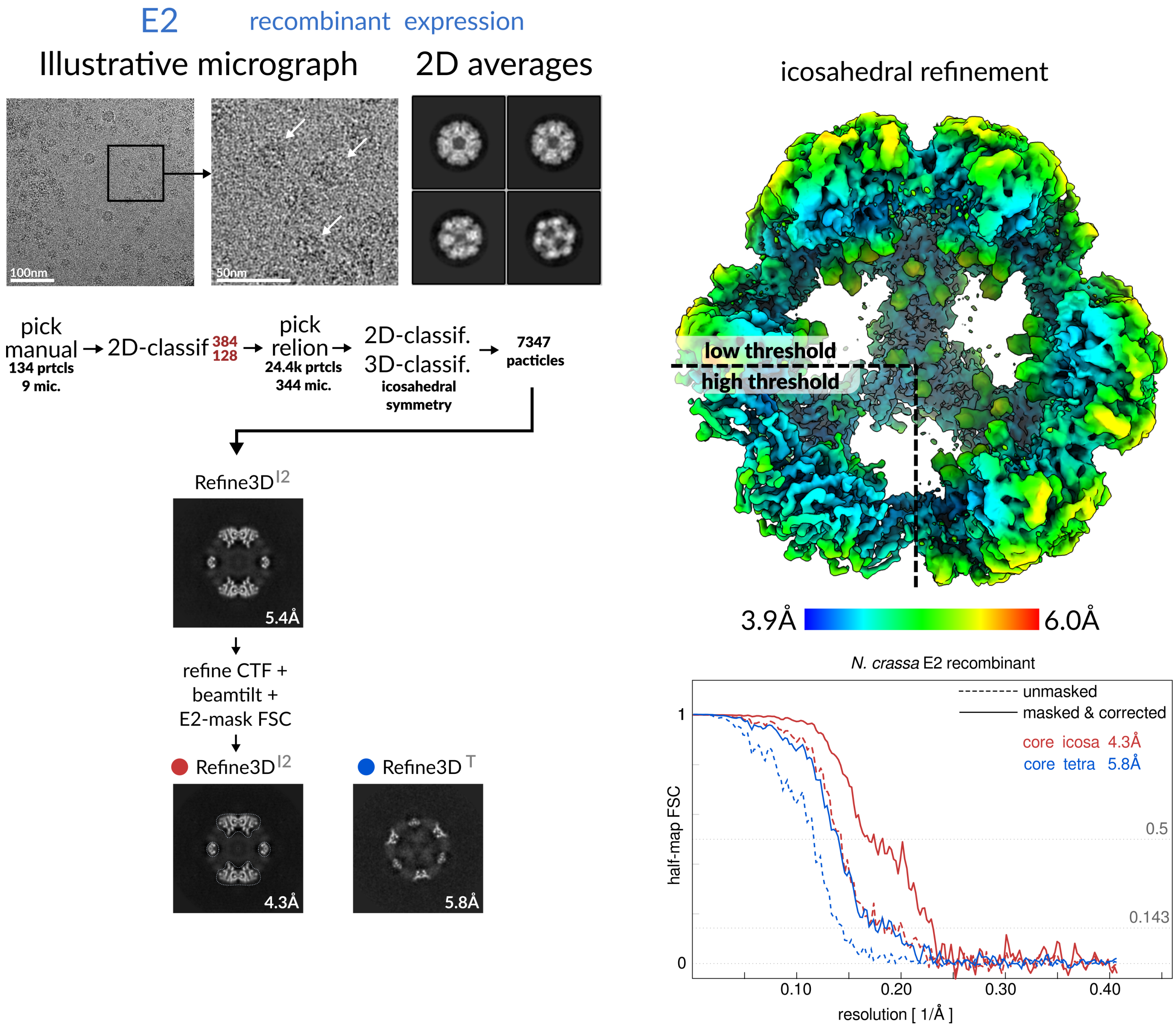

Supplementary Figure 5: **Processing of the E2 core assembly.** Corrected FSC refers to the adjustment of the half-map according to over-fitting identified by phase-randomization beyond FSC=0.8

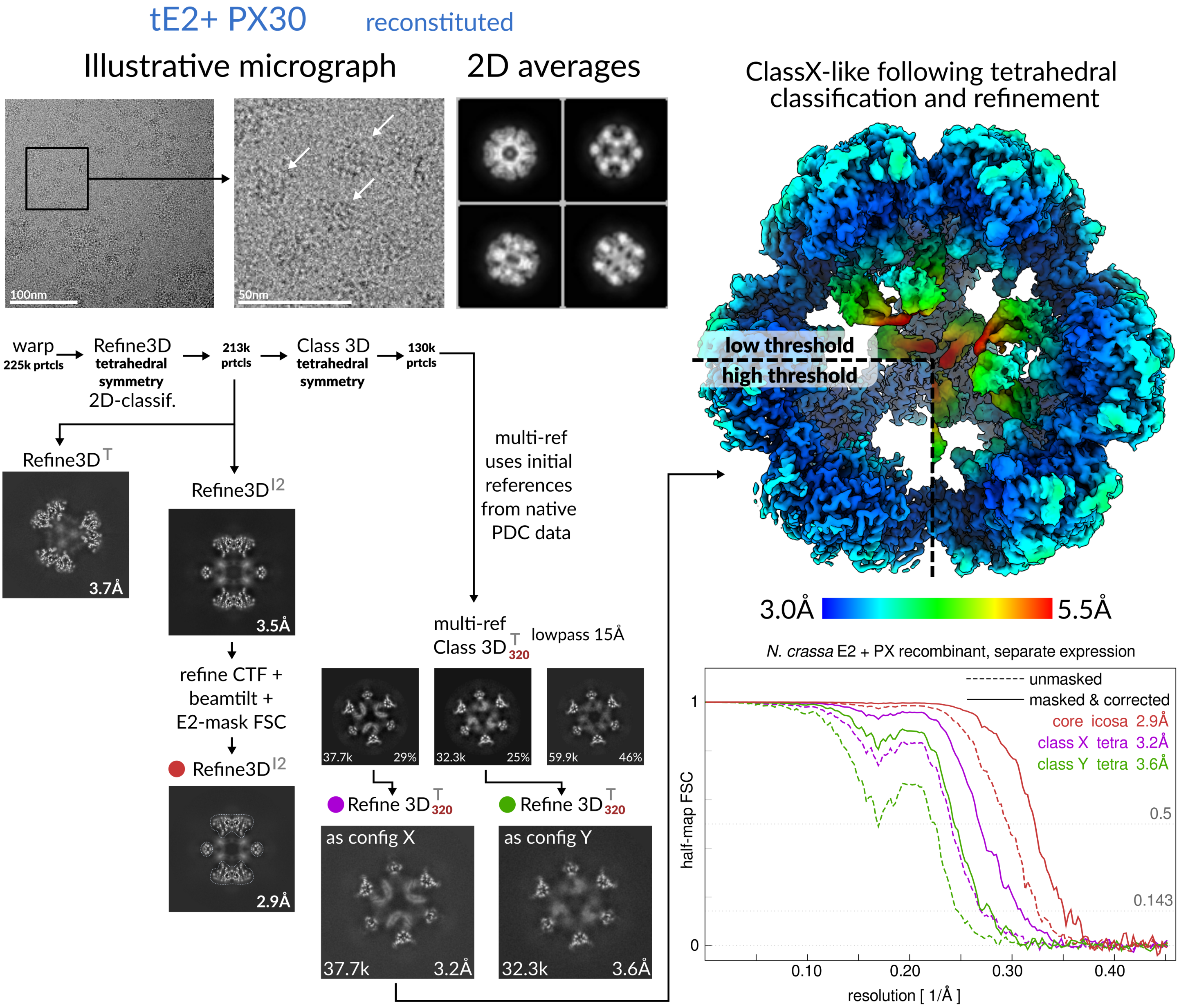

Supplementary Figure 6: **Processing of E2+PX subcomplex.** Multi-reference classification was conducted by providing three input references, corresponding to the three indicated class reconstructions X, Y and N from the native PDC classification. This introduces a measure of reference-bias. The full dataset (70k particles) was thus re-classified to assess the distribution among classes, but no inference was made from this distribution. It is shown here for completeness and to indicate the correspondence of the interior density with PX trimers. It should not be taken to indicate correspondence of the interior arrangement with configurations X or Y as a whole. Corrected FSC refers to the adjustment of the half-map according to over-fitting identified by phase-randomization beyond FSC=0.8

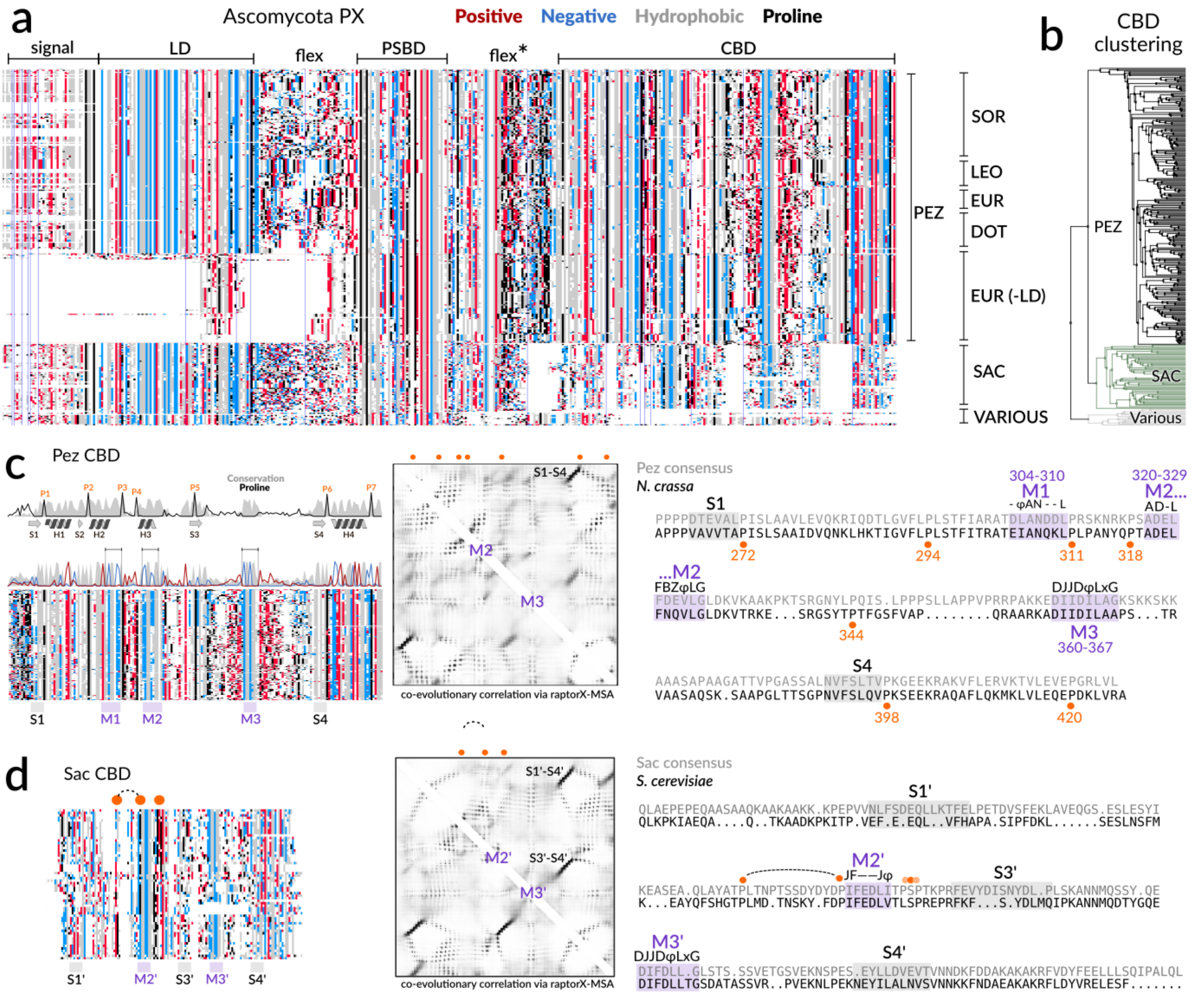

Supplementary Figure 7: **Multiple sequence alignemnts of fungal PX. a**, The overall multiple sequence alignment (MSA) of fungal (ascomycota) PX, as found by fetching all PFAM sequences annotated as E3BD-containing (the current annotation for the PSBD), filtered to exclude any E2-like core-forming protein. The PSBD-CBD linking region (indicated by an asterisk) is presumed to be flexible, but it exhibits significant sequence conservation. This may indicate a larger E3BD and reduced E3 flexibility compared to mammals, but this was not investigated further, given the rudimentary phylogenetic analysis. Three-letter labels (SOR/LEO/EUR/DOT) refer to classes within the subdivision Pezizomycotina (PEZ). Notably, the lipoyl domain (LD) is conserved but missing in some EUR sequences. The LD is not well-aligned in the aberrant sequences (labeled as various), casting doubt with regards to their purpose and/or annotation. **b**, Clustering of the isolated CBD shows PEZ and Saccharomycotina (SAC) as fundamental clusters. **c**, Isolating and re-aligning the Pez CBD (incl. *N. crassa*) shows strong conservation, especially of seven prolines that appear to delimit the overall domain topology. A co-evolutionary correlation (raptorX) using this MSA finds support for at least one long-range anti-parallel beta-sheet, supported by secondary structure prediction. There are also three similar and conserved negative motifs, M1-3. The consensus motifs are shown (J=I/L, B=D/N, Z=Q/E, $-$=D/E, ϕ=hydrophobic, x=Any). It is also notable that the region spanning P5 and P6 has low conservation but high charge and proline content. This region also contains M3. The PEZ consensus sequence is aligned to *N. crassa*, mapping P1-7 and the conservation motifs. Notably for *N. crassa*, M1 is deficient in the predicted negative charge and the P5-P6 region has surprisingly low proline-content. **d**, the SAC CBD shows lower conservation of both proline and overall residues, with the notable exception of the DJJDϕLxG-motif, which is shared with PEZ. A similar motif (JF$--$Jϕ) is also found, which does not have an obvious correspondence to either of the other motifs found in PEZ, but which has an overall correspondence with M2.

```
P16451_PX_YEAST -----GKIP--------------------------------QDSVNKVTEFIKKNERLDL 233
O00330_PX_HUMAN QLKQTGKITESRPTPAPTATPTAPSPLQATAGPSYPRPVIPPVSTPGQPNAVGTFTEIPA 280
Q7RWS2_PX_NEUCR ------------PAPPPVAVVTAPISLSAAIDVQ-----------NKLHKTIGVFLPLST 297
                                                                 : :     :

P16451_PX_YEAST SNIKPIQLKPKIAEQAQTKAADKPKITPVEFEEQ--LVFHAPASI-------PFDKLSES 284
O00330_PX_HUMAN SNIRRVIA-KRLTESKS--------TVPHAYATADCD-LGAVLKVRQDL---VKDDIKVS 327
Q7RWS2_PX_NEUCR FITR---A-TEIANQKL--------PLPANYQPTADELFNQVLGLDKVTRKESRGSYTPT 345
                   :      .:::.            *  :       :     :         .. . :

P16451_PX_YEAST LNSFMKEAYQFSH----------------GTPLMD------TNSKYFDPIFEDLV----- 317
O00330_PX_HUMAN VNDFIIKAAAVTLKQMPDVNVSWDGEGPKQLPFIDISVAVATDKGLLTPIIKDAAAKGIQ 387
Q7RWS2_PX_NEUCR FGSFVAPQRA-----------------ARKADIIDILAAPSTR--VAASAQSKSAAPGLT 386
                ...*:                           ::*      *         .. .

P16451_PX_YEAST -------TLSPREPRFKF---SYDLMQIPKANNMQDTYGQEDIFDLLTGSDAT---ASSV 364
O00330_PX_HUMAN EIADSVKALSKKARDGKLLPEEYQGGSF--SISNLGMFGIDEFTAVINPPQACILAVGRF 445
Q7RWS2_PX_NEUCR -------------------------------TSGPNVFSLQ------------------- 396
                                               .  . :. :

P16451_PX_YEAST RPVEK-------NLPEKNEYILALNVSVNNKKFNDAEAKAKRFLDYVR-ELESF------  410
O00330_PX_HUMAN RPVLKLTEDEEGNAKLQQRQLITVTMSSDSRVVD--DELATRFLKSFKANLEN-PIRLA-  501
Q7RWS2_PX_NEUCR -----VPKSEEK--------------------------RAQAFLQKMKLVLEQEPDKLVRA 426
                                                       *  **. .:  **.
```

Supplementary Figure 8: **Multiple sequence alignments of human and fungal PX.** Fungal and human PX are similar in their specific recruitment of E3. Excluding their substrate-carrier (lipoyl) domains and E3-binding domain, the sequence identity is however extremely low. We show this here by a multiple-sequence alignment including the C-terminal domain of *N. Crassa*, *S. Cerevisiae*, as well as human PX/E3BP. This alignment shows that very few points of conservation or similarity are evident, and then only by introduction of large gaps in the alignment. The highly conserved fungal motif M3 (cf. Supplementary Fig. 7, panel C+D) is indicated in bold for *N. Crassa* and *S. Cerevisiae*.

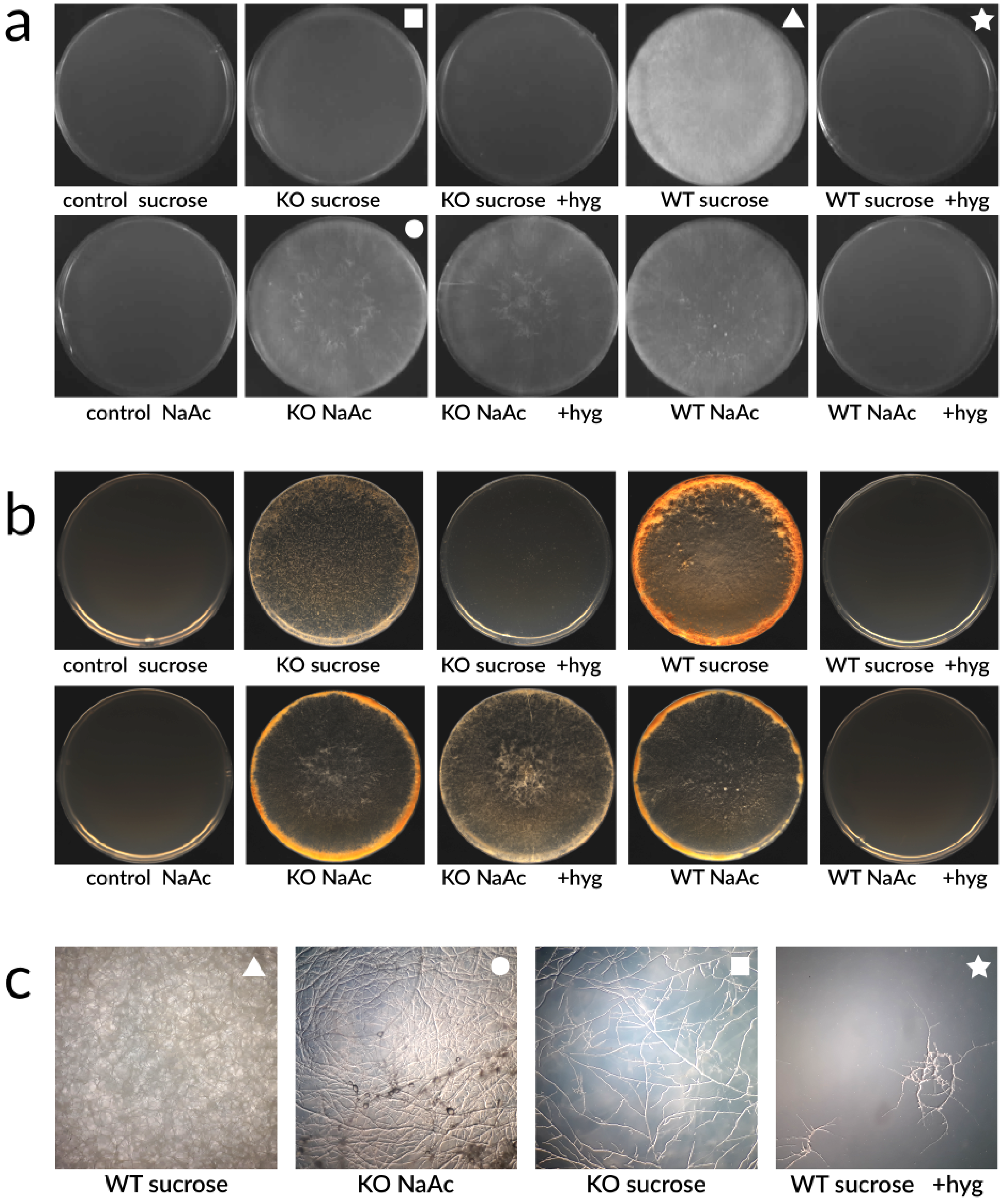

Supplementary Figure 9: **Differential growth assay results.** **a**, During 72h in darkness, mycelia of *N. crassa* wildtype grows better on 1% sucrose than on 1% Na-Acetate (NaAc) (minimal Vogel's media). Growth of a PX knockout is comparable to wildtype on NaAc, but is severely impeded on sucrose, indicating that PX is essential for pyruvate metabolism conveyed by the PDC. The knockout strain is engineered to be hygromycin-resistant, and supplementing with additional 300 μM hygromycin is lethal to WT but has a minor effect on KO growth. **b**, Following an additional 72h in light, sporulation is also comparable in knockout and wildtype grown on NaAc. Sporulation is however distinctly different on sucrose. It appears that knockout of PX is detrimental but not lethal to *N. crassa*. **c**, Mycelial growth displays an indistinguishable phenotype despite the distinct differences in growth rate.

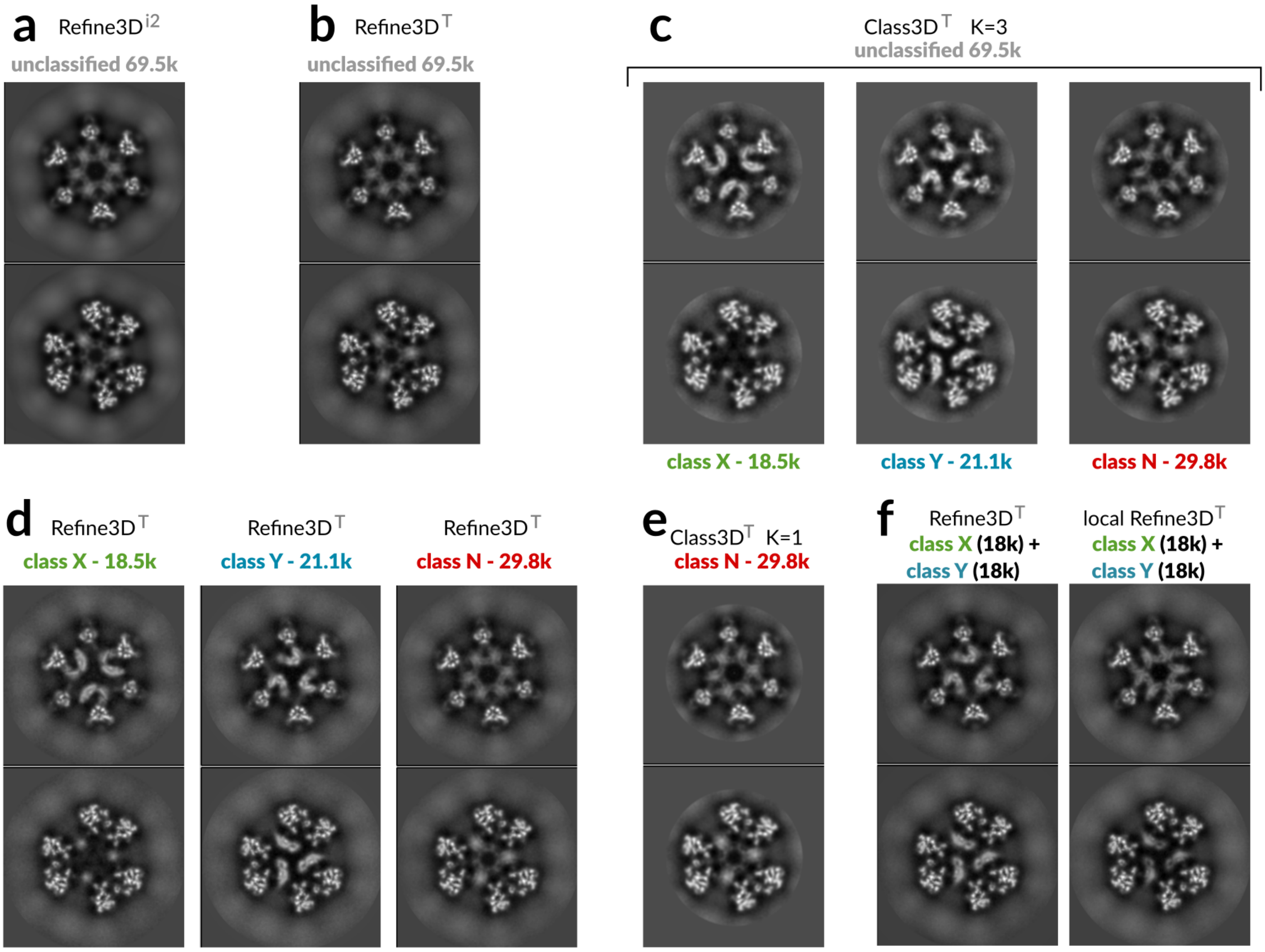

Supplementary Figure 10: **Reconstruction of recombined classes X and Y. a**, Refinement of the endogenous PDC as icosahedral shows over-symmetrization of a core-interior component. **b**, Implicit symmetrization and non-tetrahedral particles cause the refinement of PDC as tetrahedral to be similarly over-symmetrized prior to classification. **c**, Tetrahedral classification is however able to show distinct classes that do not superimpose (X and Y), as well as one still over-symmetrized class (N). **d**, Refinement of class N as tetrahedral is similar but not identical to the classified reconstruction, indicating that some feature of the data in subclass N is hidden during classification (see also section 1.5). **e**, A classification of subclass N with a single class circumvents the use of local angular searches around a single orientation in the final steps of refinement, and does resemble the original classification. **f**, Refinement of a re-combined dataset containing equal proportions of X and Y arrangements (18k each), produce a Y-like reconstruction (compare inset to panel **d**), rather than a superimposed X/Y reconstruction. This shows efficient hiding of the X-classified data by weighted backprojection. By enforcing only local alignment to be used, data hiding by weighted back projection is prohibited, and the superimposed class X and Y is then also recovered in line with expectation. Note that no previous reconstruction shows a similar superposition. Local alignments in this case start from those found in individual X and Y refinements respectively.

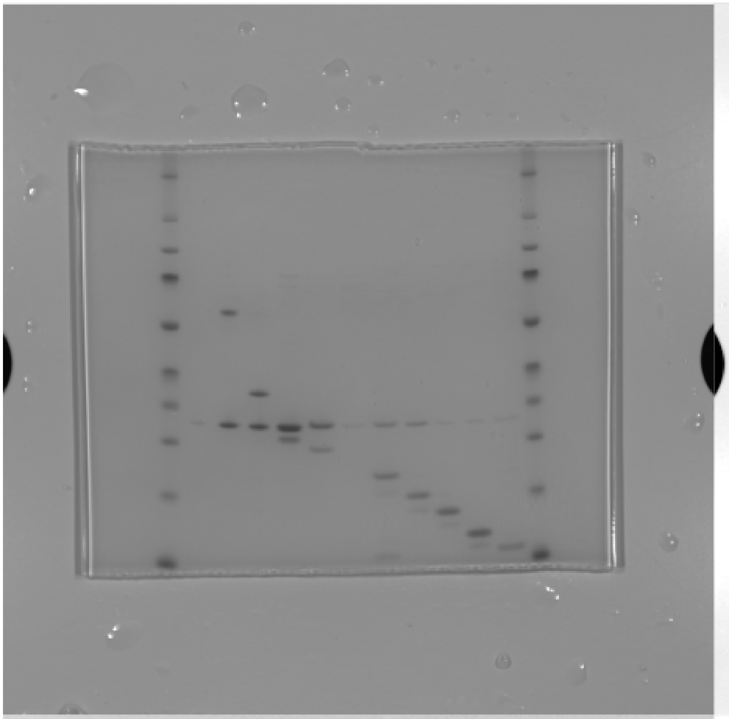

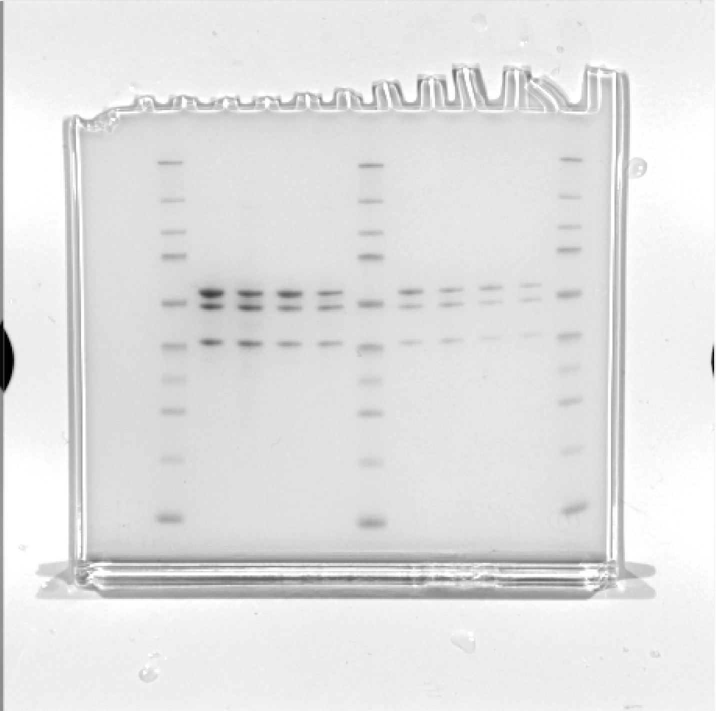

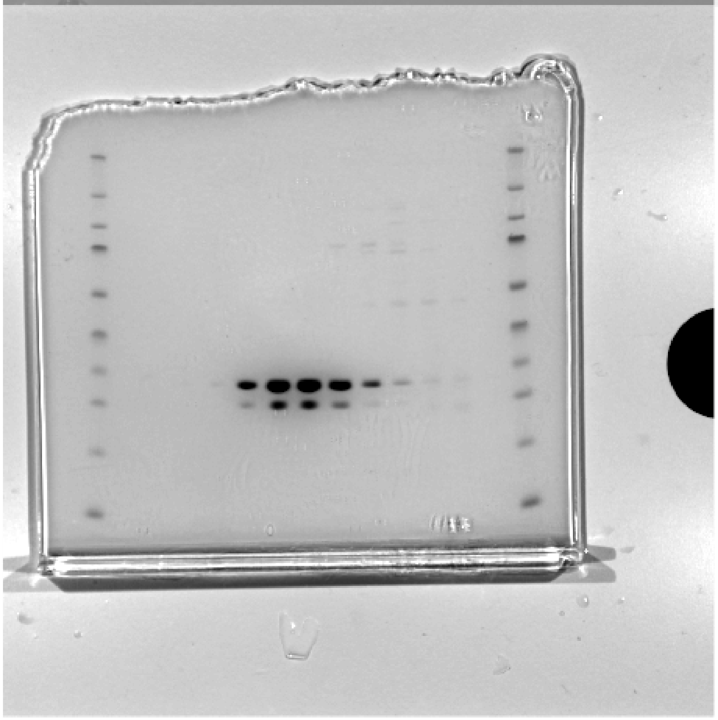

Supplementary Figure 11: **Unadulterated gel photos** Used in Fig. 3 and Supplementary Fig. 4
